# Application of transposon insertion site sequencing method in the exploration of gene function in microalgae

**DOI:** 10.3389/fmicb.2023.1111794

**Published:** 2023-02-03

**Authors:** Xiaobing Hu, Yulong Fan, Chengfeng Mao, Hui Chen, Qiang Wang

**Affiliations:** ^1^State Key Laboratory of Crop Stress Adaptation and Improvement, School of Life Sciences, Henan University, Kaifeng, China; ^2^School of Environmental Engineering, Yellow River Conservancy Technical Institute, Kaifeng, China; ^3^Academy for Advanced Interdisciplinary Studies, Henan University, Kaifeng, China

**Keywords:** microalgae, chassis cell, transposon, flanking sequence, sequencing

## Abstract

Microalgae are a large group of organisms that can produce various useful substances through photosynthesis. Microalgae need to be genetically modified at the molecular level to become “Chassis Cells” for food, medicine, energy, and environmental protection and, consequently, obtain benefits from microalgae resources. Insertional mutagenesis of microalgae using transposons is a practical possibility for understanding the function of microalgae genes. Theoretical and technical support is provided in this manuscript for applying transposons to microalgae gene function by summarizing the sequencing method of transposon insertion sites.

## 1. Introduction

Microalgae are one of the oldest groups of organisms on Earth, contributing more than 50% of the primary productivity of the entire planet (Sun et al., 2022). Compared with other biomass resources, microalgae occupy cultivated lands, have high biomass, grow at a fast rate, have high adaptability, are easy to domesticate, and have high light energy utilization. Additionally, through genetic transformation, engineered microalgal chassis cells can fix CO_2_ (Singh and Ahluwalia, 2012) through photosynthesis to produce substances, including oils, proteins, amino acids, polysaccharides, and vitamins. Currently, microalgae have been widely used in food (Scieszka and Klewicka, 2019; Fu et al., 2021), medicine (Beaumont et al., 2021), energy (Zhang et al., 2012; Frigon et al., 2013; Bigelow et al., 2014), environmental protection (Cabanelas et al., 2013; Gomez et al., 2013; Chen W. et al., 2016), feed (Vidyashankar et al., 2014; Packer et al., 2016), and other fields. Therefore, these organisms gradually became critical raw materials for the active extraction of substances (Liu et al., 2022). Engineering microalgal chassis cells will become an effective force in achieving the goal of carbon neutralization worldwide and, consequently, replacing traditional industries.

It is essential to further understand the gene functions of microalgae in depth to utilize microalgae resources. However, the gene functions of a considerable proportion of microalgae remain unknown. More methods are being used to analyze and identify gene functions with the continuous development and innovation of new molecular biology technologies and methods (Ng et al., 2020). The functional genomics sub-discipline gradually formed after such approaches were developed. The construction of effective mutants is an essential method in functional genomics research. There are many methods for obtaining mutants of genes, and transposons to construct mutants have a random nature. This method may better understand gene functions and the connections between related genes (van Opijnen and Camilli, 2013).

Transposons are mobile DNA genetic sequences that can “jump” to distinct locations in the genome and are found in prokaryotic and eukaryotic genomes (Choi and Kim, 2009). Barbara McClintock discovered the first transposon in maize (Gierl and Saedler, 1992). Transposon tags have long been considered a powerful research tool for randomly distributing primer binding sites, generating mutations, and introducing physical or genetic tags into large target DNA (Damasceno et al., 2010; Shapiro, 2010). Therefore, the random insertion of transposon mutations is a desirable choice, especially if one wants to create many mutants.

After the insertion of a mutation is completed in the transposon, the gene identification and location of the insertion mutation must be solved (Li et al., 2020). This means understanding the gene sequences on either side of the insertion site of the mutant by sequencing. We can only understand the function that a gene may have through the correlation between the mutation position and the phenotype. Therefore, standard sequencing methods involving microalgae are introduced and summarized in this study.

## 2. Enzymatic digestion

Restriction endonucleases are used in the enzymatic digestion method to digest the microalgae genome before amplification and sequencing. This method is straightforward, has low costs, and is easy to operate, but the success rate is low. It is suitable for mutants with a negligible overall genome and appropriate restriction endonucleases. Standard methods are described as follows.

### 2.1. Reverse PCR

**Figure 1:**
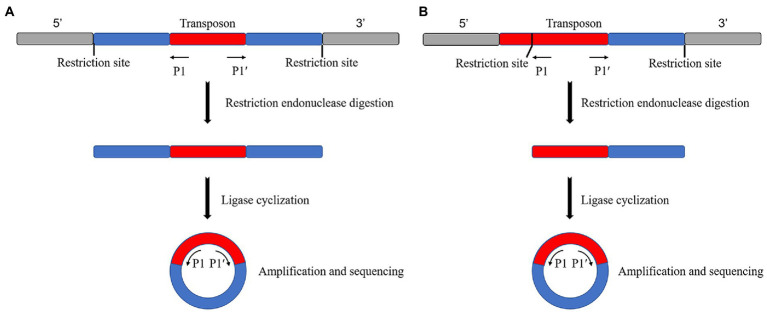
Schematic diagram of the reverse PCR principle. **(A)** No restriction endonuclease digestion site on the transposon, sequenced as both sides of the transposon insertion site after self-associative cyclization. **(B)** Single restriction endonuclease digestion site on transposon, sequenced as a unilateral sequence of the transposon insertion site after self-associative cyclization.

Reverse PCR is used to find a restriction endonuclease with more enzymatic sites and broader distribution in the mutant genome, but no enzymatic sites or only one enzymatic site in the transposon sequence or fragment the genome by enzymatic digestion. The DNA fragment is self-associated after enzymatic digestion by ligase to cyclize it. Specific primers can be designed from the transposons if the cyclized genome contains transposons. Specific primers can be designed from the transposon for amplification and sequencing to obtain the transposon insertion site if the transposon is included in the genome. The sequence obtained is on both sides of the insertion site if there is no enzyme cut site in the transposon (Figure 1A). Finally, the sequence obtained is on one side of the insertion site if there is a single enzyme-cut site (Figure 1B).

The reverse PCR method is simple in principle and operation, and its experimental cost is low. However, it is unsuitable for high-throughput sequencing. Additionally, it is not stable as a sequencing method because of its specific requirements for selecting restricted endonucleases and restrictions on the transposon insertion position. In addition, its sequencing length is not fixed. Feng et al. (2008) used reverse PCR to amplify the 5′ and 3′ flanking sequences of the TaCKX1 gene. These researchers obtained the full-length DNA sequence of TaCKX1 by cloning the TaCKX1 fragment from a conserved sequence of wheat cytokinin oxidase/dehydrogenase (CKX). Wang et al. (2021) used reverse PCR to amplify and detect the FSTA gene in transgenic zebrafish genomic DNA. This method may be used for gene doping detection in blood samples or to assess the safety of gene therapy and GMOs. Su et al. (2021) used this assay for transposase-accessible chromatin using the sequencing (ATAC-seq) method combined with the “Circle_finder” bioinformatic algorithm to predict extrachromosomal circular DNA (eccDNA) in human cancer cells. These researchers validated the detection of eccDNA using reverse PCR. Gutiérrez et al. (2021) used reverse PCR to identify chronic myeloid leukemia (CML) at the genomic level with the breakpoint sequence of the signature fusion gene *BCR-ABL1*. They applied this method to seven real cases.

### 2.2. Plasmid rescue

**Figure 2:**
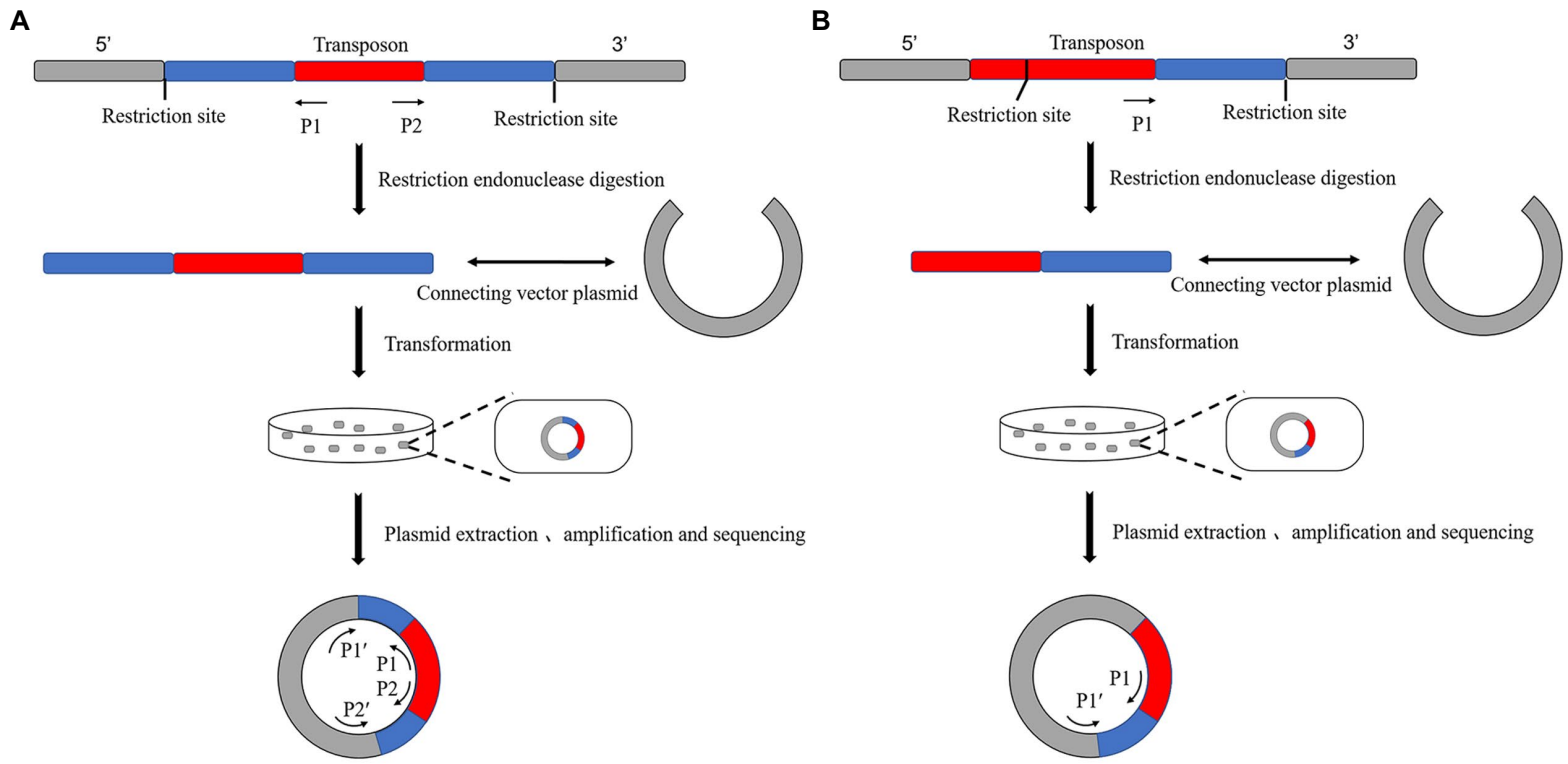
Schematic diagram of the principle of plasmid rescue. **(A)** There is no restriction endonuclease site on the transposon. The vector plasmid was ligated after enzymatic digestion and transformed into *E. coli*. The positive new bacteria were obtained by selective labeling. The plasmid is extracted, and the sequence of both sides of the transposon insertion site can be measured by amplifying and sequencing two pairs of specific primers on the transposon and plasmid. **(B)** There is a single restriction endonuclease site on the transposon. The plasmid was extracted from a positive bacterium obtained using selective labeling. The unilateral sequence of the transposon insertion site could be sequenced by amplifying and sequencing the transposon with a pair of specific primers on the transposon and plasmid.

The principle of plasmid rescue is similar to reverse PCR. The standard operation is (1) to insert the transposon into the genome, (2) digest it with a restriction endonuclease, (3) ligate it into a cloning vector, (4) transform it into an *Escherichia coli* culture, (5) select positive bacteria according to the label carried by the plasmid, (6) culture it, (7) extract its plasmid, and (8) sequence it according to the specific primers of the transposon and plasmid (Tsurumaru et al., 2008; Figure 2).

The advantage of the plasmid rescue method is the high specificity of the fragments obtained. The disadvantages of this method are the requirement for the selection of endonucleases, the lack of experimental stability, and the unsuitability for large-scale high-throughput sequencing. Huang et al. (2009) used a combination of plasmid rescue and reverse PCR to simultaneously determine the sequences on both sides of the *Drosophila* P-transposon insertion site. Kemppainen et al. (2008) randomly determined the genomic DNA sequences of the T-DNA right border (Rb) of 51 strains from a considerable number (~500) of T-DNA insertion mutants of *Laccaria bicolor* using plasmid rescue. Sixty-nine percent of the flanking sequences of this species were successfully determined. At the same time, 87% of these sequences were successfully localized in the genome.

### 2.3. Specific enzymatic cleavage

**Figure 3:**
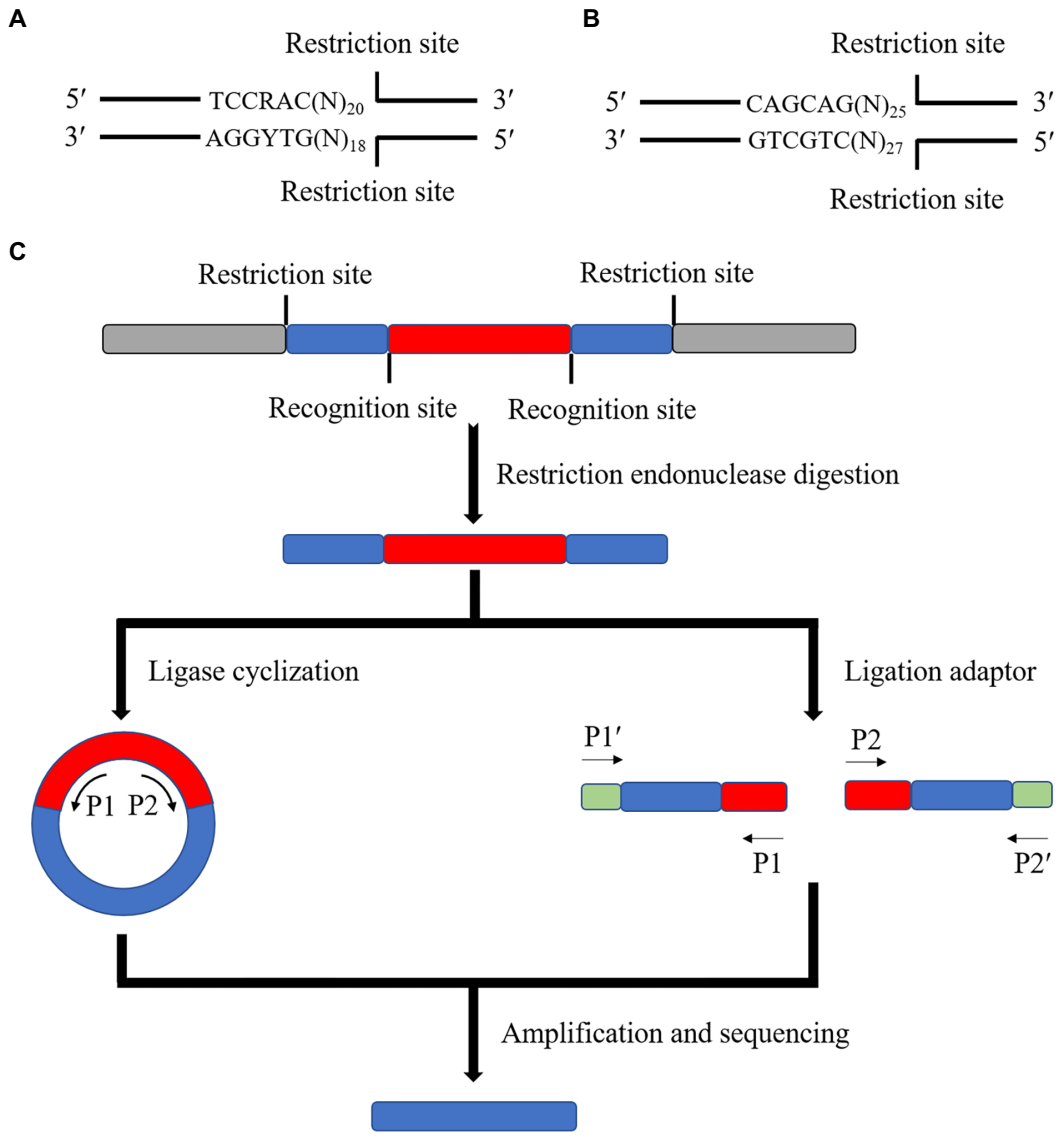
Schematic diagram of the principle of the special enzyme method. **(A)** Schematic diagram of the MmeI enzyme cleavage site (R is any purine, Y is any pyrimidine, N is any base). **(B)** Schematic diagram of the EcoP15I enzyme cleavage site. **(C)** The special enzyme cleavage method is to modify both ends of the transposon in advance and load the recognition site of a particular enzyme before transposition. After inserting the transposon into the target genome, the sequence of 18 ~ 27 bp is left on both sides of the fragment containing the transposon using a special enzyme cleavage. The ligase is cyclized or connected to the sequencing junction to amplify and sequence with the specific primer or junction sequence on the transposon.

Some specific restriction endonucleases are used in this method. These endonucleases have a common point: the enzymatic cut site is located after several bases of the recognition site. Therefore, a segment of the base sequence of the recognition site can be left after the enzymatic cut. For example, MmeI (Figure 3A) and EcoP15I (Figure 3B) can retain about 18 ~ 27 bp after the recognition site after enzymatic cleavage. Therefore, these enzymatic cleavage sites can be inserted at both ends of the transposon. The insertion site was sequenced by amplifying the specific sequences on the specific motifs by enzymatic ligation or by directly ligating special connectors. Finally, the insertion site was found against the target genome (Figure 3C).

The advantage of this method is that it is simple. Only particular enzyme cleavage sites must be added on both sides of the transposon. It can support high-throughput large-scale sequencing by connecting Illumina adapter sequences. The disadvantage of this method is related to the short localization of the sequence, which is only about 38~52 bp. Therefore, sometimes even the genome length that can be measured is less than 38 bp to consider joint connection and sequencing problems. The specificity of the method is weak, and the position of the transposon insertion mutation cannot be accurately determined in some genomes with more repetitive sequences or palindromic sequences. Additionally, the particular enzyme cleavage method cannot be used for transposons, in which transposase recognition sites are at both ends because the enzyme cleavage sites cannot be added.

Regarding the application of the method, Ng et al. (2005) investigated a more accurate and efficient way of determining cDNA using the MmeI endonuclease in conjunction with other common endonucleases. These researchers mapped the cDNA to genomic sequences to delineate the transcriptional boundaries of each gene. Matsumura et al. (2003) used the EcoP15I endonuclease to analyze the sequence of cDNA applied to monitor the genome sequences of rice and *P. aeruginosa*. These researchers found that hydrophobic protein genes were the most actively transcribed in *P. aeruginosa* leaves. They also studied gene expression changes in *Benthamiana*, a model organism, before the hypersensitive response induced by INF1, allowing the rapid identification of genes that were up- or down-regulated by the induction. Zhang et al. (2014) investigated a method to determine the transcriptional boundaries of a high-throughput sequencing method for determining transposon insertion sites in *Chlamydomonas reinhardtii*. The species was investigated and applied to a mutant library, and 11,478 insertion sites were identified.

## 3. Multiple primer amplification method

The multiple primer amplification methods are developed based on chromosome stepping and nested PCR principles. Nested PCR is a multiple primer PCR method designed to enhance the specificity of the pairing between primers and templates based on standard PCR. The principle is straightforward. The most basic nested PCR is to set two sets of PCR primers for two rounds of PCR amplification using the first pair of primers (also known as external primers) for multiple cycles of standard amplification of the target DNA. Part of the amplified product is diluted after the first amplification round and used as a template for the second round of amplification, using the second pair of primers (known as internal primers or nested primers, combined with the first round of PCR products). The second primer pair, called internal primers or nested primers, which are combined inside the PCR product of the first round, is used for multiple amplification cycles. Sometimes, a third or fourth primer pair can be used for amplification, depending on the experiment.

However, when designing primers, specific primers in nested PCR are not designed based on randomly inserted transposons. Two PCR rounds can only be completed after some universal primers are created, which often cannot be used directly in the practical application of transposon insertion mutant sequencing. Therefore, some improved methods have been derived and are described below.

### 3.1. Thermal asymmetric interleaving PCR (TAIL PCR)

The basic principle of TAIL PCR is the same as nested PCR. TAIL PCR is based on designing multiple sets of nested specific primers with a higher annealing temperature (Tm) on the transposon and a shorter and lower Tm value of random simpler primers. The target sequence was amplified by amplifying different specific primers and simpler primers using the difference in Tm values (Liu et al., 1995; Liu and Wittier, 1995).

**Figure 4:**
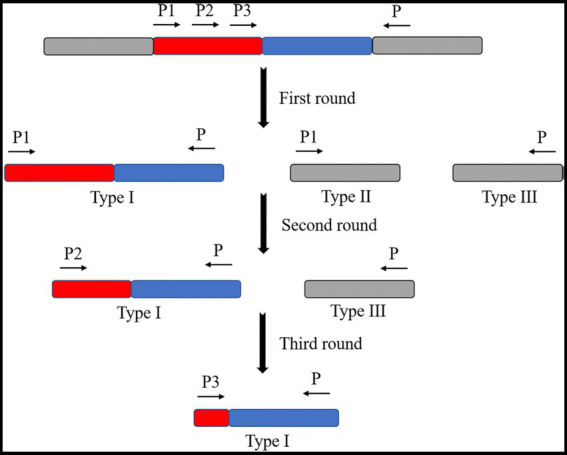
Schematic diagram of the TAIL-PCR principle. Three specific primers were designed on the transposon. One randomly abridged primer is designed to produce three products by the first amplification cycle (high specificity reaction, low specificity reaction, low specificity reaction, and thermally asymmetric super response). These are the target fragment (type I product), the fragment amplified by the specific primer itself (type II product), and the fragment amplified by the abridged primer itself (type III product). The target fragment was selectively amplified by the second amplification cycle (thermally asymmetric super reaction). The target fragment was further amplified in the third amplification cycle (thermally asymmetric super reaction or normal PCR reaction).

The commonly used TAIL PCR amplification method consists of three cycles of PCR reactions (Table 1; Liu and Huang, 1998). In the first cycle, products amplified by a specific primer one and simplex primer are yielded by the PCR reaction (type I). The products were amplified by a particular primer (type II). The products are amplified by a simplex primer (type III). During the second PCR reaction cycle, the product of the first cycle was diluted as a template. The product of the type I primer is selectively amplified using specific primer two. This primer was made using a simplex primer in a thermally asymmetric supercycle. The product of the second PCR reaction cycle was diluted in the third PCR reaction cycle. A template and a specific primer from the third cycle with a simplex primer are used in a normal PCR reaction cycle or a thermally asymmetric supercycle. The target fragment was further amplified to obtain the sequence on one side of the transposon insertion site (Figure 4).

**Table 1:** TAIL PCR amplification procedures.

Reaction	Procedures		Products
	Number of cycles	Reaction conditions	
First PCR cycle	1	92°C (180 s), 95°C (60 s)	The target sequences amplify linearly by annealing and extending specific primer one with transposon sequences at high annealing temperatures. At the same time, the concentration of non-specific products resulting from the binding of the simplex primers is low.
	5	94°C (30 s), 65°C (60 s), 72°C (120 s)	
	1	94°C (30 s), 25°C (120 s), not less than 120 s to 72°C, 72°C (120 s)	The low annealing temperature allows better binding of the simplex primers to a larger number of target sequences.
	10	94°C (30 s), 44°C (60 s), 72°C (120 s)	The lower annealing temperature allows both primers to anneal to the template. Thereby, it enables the original single-stranded target DNA produced by the high specificity cycle to be replicated into double-stranded DNA in preparation for the next round of amplification.
	12	94°C (30 s), 65°C (60 s), 72°C (120 s), 94°C (30 s), 65°C (60 s), 72°C (120 s), 94°C (30 s), 44°C (60 s), 72°C (120 s)	Alternating cycles of higher and lower annealing temperatures allow exponential amplification of the target fragment. This exceeds the amount of non-target fragments.
	1	72°C (300 s)	
Second PCR cycle	12	94°C (30 s), 65°C (60 s), 72°C (120 s), 94°C (30 s), 65°C (60 s), 72°C (120 s), 94°C (30 s), 45°C (60 s), 72°C (120 s)	The first round of product dilution is used as a template. Then, the specific primer two and the combination of the simplex primers allow the specific product to be selectively amplified.
	1	72°C (300 s)	
Third PCR cycle	20	94°C (40 s), 45°C (60 s), 72°C (120 s)	After the second round of product dilution as the template, the specific primer three was combined with the simplex primer. The target fragment was further specifically amplified to obtain the target sequence flanking the transposon.
	1	72°C (300 s)	

The main advantage of the TAIL PCR method is that it does not require DNA manipulation before PCR. Cyclization and ligation are avoided and have a faster reaction speed, higher specificity, and higher efficiency. However, nonspecific binding due to low temperature can still exist and may sometimes lead to amplification and sequencing failures or situations where the amplified sequence length is insufficient. Various improved versions of amplification protocols are constantly updated as TAIL PCR continues to develop. For instance, the success rate and amplification sequence length are improved by the method by setting multiple sets of more extended simplex primers, increasing the success rate to 90% and the amplification sequence length to 1–3 kb (Liu and Chen, 2007).

Yuan et al. (2009) used a combination of TAIL PCR and plasmid rescue to efficiently identify eight insertional mutation sites in a library of rice streak transposon Tn5 insertional mutants with attenuated virulence on rice. These researchers used this method to determine the corresponding functional genes efficiently. Oranab et al. (2021) used the TAIL PCR technique to examine the T-DNA insertion sites of the activation marker mutants of the CNGC19 and CNGC20 genes in *Arabidopsis* cyclic nucleotide-gated ion channels (CNGCs) under salt stress conditions. Thus, it lays the groundwork for studying the role of CNGC19 and CNGC20 in *Arabidopsis* under salt stress regulation. Wang et al. (2013) used a modified TAIL PCR technique to examine the genome of *Wolbachia*. The WO genome of the mild phage on *Wolbachia* was determined using a modified TAIL PCR technique. The evolution of the WO genome was also assessed by comparing the WO genomes of infested fig wasps with those of infected insects. The following species were considered: the pink spotted borer moth, *Culex* mosquito, *Drosophila melanogaster*, *Drosophila anthropomorphis*, and the lyre fly nymphal set of golden wasps.

### 3.2. Rapid amplification of cDNA ends (RACE)

RACE is a technique based on reverse transcription PCR to rapidly amplify the 5′ and 3′ ends of cDNA from samples (Chutia et al., 2020). Since cDNA differs in prokaryotic and eukaryotic algae, and the situation is different at the 5′ and 3′ ends, various amplification methods are described below.

**Figure 5:**
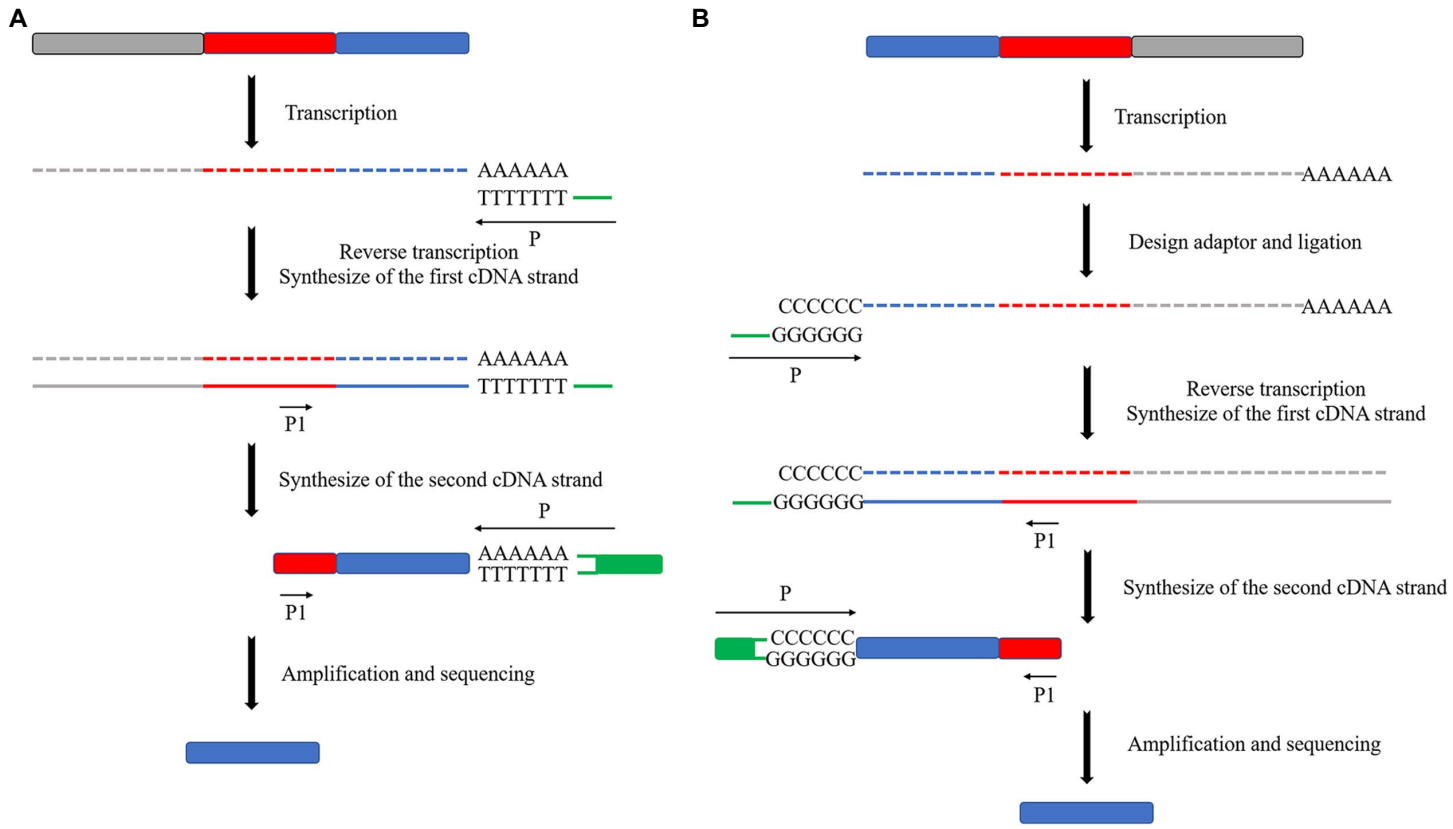
Schematic diagram of the principle of RACE in eukaryotes. **(A)** 3′ end RACE utilizes the post-transcriptional poly(A) tail structure of mRNA first to reverse transcribe the first cDNA strand containing the transposon sequence and then synthesize the second cDNA strand by using specific primers on the transposon sequence. **(B)** 5′ end RACE is performed by ligating a poly(C) tail structure after transcription. Then, the same operation as 3′ end RACE is performed.

Reverse transcription primers were designed for eukaryotic microalgae to reverse the transcription of the first cDNA strand based on the naturally occurring poly(A) tail at the 3′ end of mRNA (Passmore and Coller, 2021). Specific primers were designed to synthesize the second cDNA strand based on transposon sequences. Subsequently, PCR amplification of the obtained cDNA strand was performed with the specific primer and the 3′ end primer of the righteous strand as a pair of primers to obtain the 3′ end sequence of cDNA (Figure 5A). In contrast, it is necessary to design specific primers based on transposon sequences, since there is no naturally recognizable sequence at the 5′ end of eukaryotic microalgae mRNA. Therefore, it will be possible to reverse transcribe it to obtain the first cDNA strand. At the same time, primer sequences at the 3′ end of cDNA by enzymatic linkage will be added, often with a poly(C) tail, and specific primers will be designed to synthesize the second cDNA strand based on the added sequence. The second cDNA strand was used as a template to synthesize double-stranded cDNA using transposon-specific primers. Finally, the cDNA 5′ end sequence was obtained by PCR amplification using transposon-specific primers and antisense strand 3′ end primers (Figure 5B).

**Figure 6:**
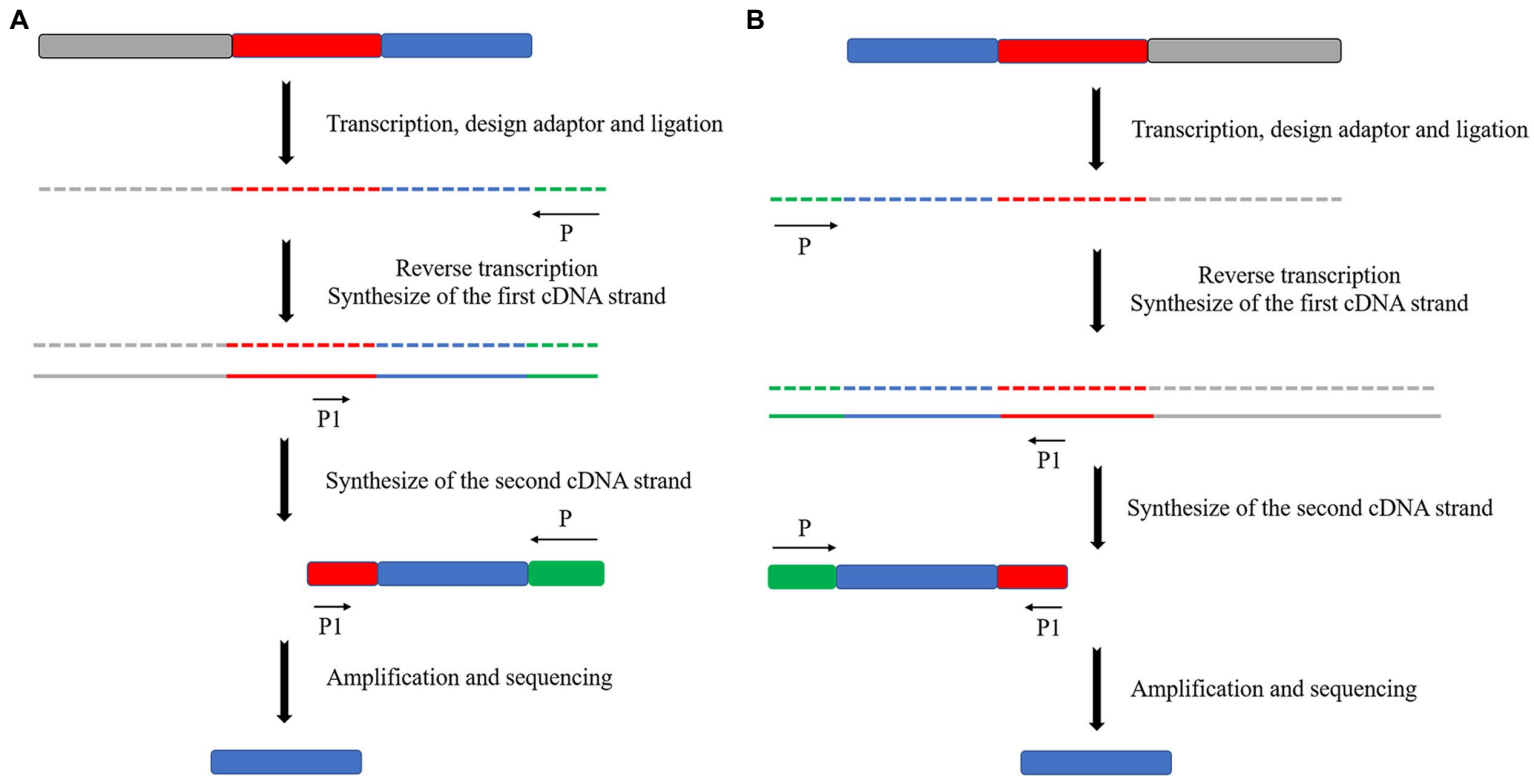
Schematic diagram of the principle of RACE in prokaryotes. **(A)** 3′ end RACE. **(B)** 5′ end RACE.

The 3′ end of mRNA does not have a special structure similar to the poly(A) tail for prokaryotic microalgae. Therefore, a splice sequence must be directly attached to the 3′ end of the mRNA to replace the poly(A) tail. The other operations are consistent with the eukaryotic microalgae 3′ end in RACE (Figure 6A). The 5′ end of prokaryotic microalgae in RACE is the same as that of eukaryotic microalgae. This requires the addition of a splice sequence at the 5′ end of the cDNA after reverse transcription and amplification (Figure 6B).

Meslet-Cladière and Vallon (2012) determined the flanking sequences of 38 randomly selected insertion mutants in a transposon insertion mutation library of the model organism *C. reinhardtii*. These authors used the 3′ end of the RACE technique. Twenty-seven (71%) were valid flanking sequences, and 23 could be accurately localized in the genome. Hu et al. (2017) identified small regulatory RNAs (SRNAs) in *Synechocystis* sp. PCC 6803 uses 5′ and 3′ ends in the RACE method, naming it RblR. RblR positively regulates the gene *rbcL*. *rbcL* encodes a large chain of Rubisco, an enzyme that catalyzes carbon fixation under different stress conditions. Thus, it affects photosynthesis regulation in PCC 6803. Li et al. (2022) determined the sequence of small antisense RNA (ThfR) on the reverse complementary strand of the *sll1414* (*thf1*) gene in PCC 6803 was used in the 5′ and 3′ ends in the RACE technique. These researchers investigated the relationship between ThfR and gene *thf1* by examining its high- and low-expression mutants.

### 3.3. Linear amplification-mediated PCR (LAM-PCR)

**Figure 7:**
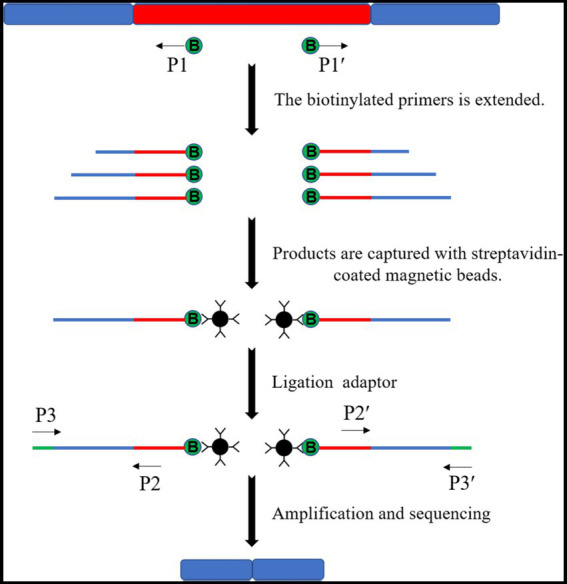
Schematic diagram of the LAM-PCR principle. Multiple single-stranded DNAs of varying lengths were amplified by the biotinylated primers. Then, multiple single-stranded DNAs are captured by streptavidin-coated magnetic beads to ligate the splice sequences and complete amplification and sequencing.

Target products are obtained in the linear amplification mediated-PCR (LAM-PCR) method by designing multiple primer sets amplified step by step. The first step is to amplify single-stranded DNA using transposon-specific primers with biotin. The amplified single-stranded DNA is captured by the adsorption of biotin by streptavidin magnetic beads. The insertion site flanking sequence was obtained by amplification and sequencing (Figure 7). This method is precise, has a high success rate, and may be designed to link Illumina junctions in the second round of amplification primers if needed (Carette et al., 2011). However, this method is more expensive for sequencing individual mutants, if not high-throughput sequencing.

Schmidt et al. (2007) used LAM-PCR to detect integration sites representing unique molecular markers for each transduced cell and its clonal progeny in the cells of an integration vector system for clinical gene therapy. Gabriel et al. (2014) used LAM-PCR to demonstrate that leukemia originated from the provirus-induced overexpression of adjacent proto-oncogenes in gene therapy patients. It was possible to bypass restriction digestion with LAM-PCR, eliminating retrieval bias at the integration site. This enabled a comprehensive analysis of the provirus location in the host genome, detailing a stepwise amplification method that integrates adjacent 3′ and 5′ sequences of the lentiviral vector.

## 4. Transposon mutagenesis coupled with next-generation sequencing (Tn-Seq)

**Figure 8:**
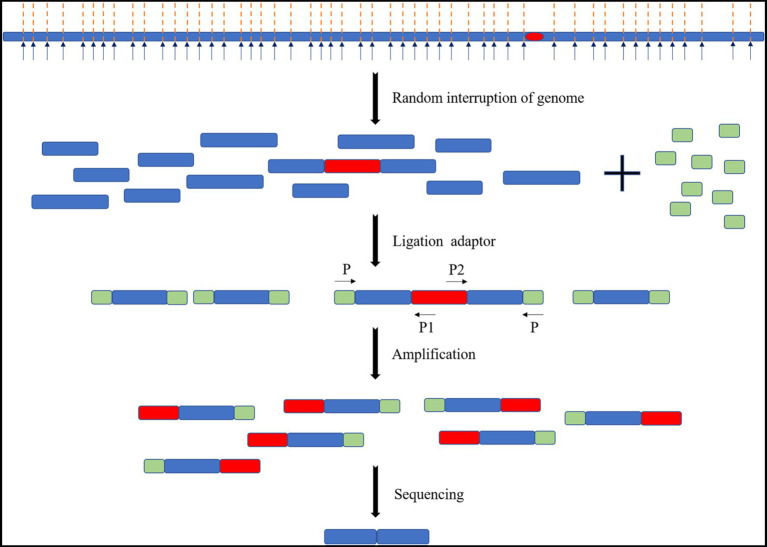
Schematic diagram of the Tn-Seq principle. The genome of the transposon insert was randomly interrupted. The splice sequence is added, and primers are designed with the specific sequences of the splice sequence and transposon for amplification and sequencing.

Transposon mutation combined with next-generation sequencing (Tn-Seq) is a high-throughput analysis method for transposon insertion. The basic idea of this method is to (1) physically or enzymatically interrupt the genome of the inserted transposon, (2) ligate the splice sequence required for next-generation sequencing to each fragment, and (3) amplify the specific sequence on one side of the transposon and the splice sequence on the corresponding side as primers. These steps were made to obtain DNA fragments of an appropriate size and perform next-generation sequencing (Figure 8). There are many other conceptually similar methods, including Tradis, HITS, INSeq, and TnLE-Seq (Wetmore et al., 2015). These methods have a common feature in that many transposon mutants are mixed. The abundance of transposon insertion into each gene may only be determined by high-throughput sequencing under certain growth conditions, such as the fitness of each gene under that growth condition, but by trying to separate the individual. However, it is difficult to isolate each mutant and match the insertion sites one by one.

Rubin et al. (2015) used transposons with molecular barcode “tags” into the genome of the prokaryotic microalga PCC 7942. They sequenced random molecular barcode transposon insertion mutation sites (RB-TnSeq) to create a library containing more than 250,000 transposon mutants and sequenced them to identify insertion sites. A total of 718 genes out of 2,723 were identified as necessary for the survival of the organism under laboratory conditions through an analysis of the distribution and survival of these mutants. Li et al. (2019) generated a mutant library of eukaryotic microalgae *C. reinhardtii* by adding a DNA barcode to transposons 3′ and 5′ respectively through RB TnSeq. The library has 62,389 mutants and covering 83% of the nuclear protein-coding genes. A genome-wide survey of genes required for photosynthesis identified 303 candidate genes. Of these, 21 of the 43 high-confidence genes were newly identified and relevant for photosynthesis.

## 5. Application of the transposon insertion site sequencing method in microalgae

Microalgae are considered significant renewable biological resources as the mainstay of photosynthesis on Earth. Certain algae have high biomass, short growth cycles, are easy to culture, and have a high content of valuable substances. Using transposons to insert mutations into microalgae genes and sequencing insertion sites to understand insertion locations and genes to determine microalgae gene functions and between-gene interrelationships are standard methods in this biological group.

**Figure 9:**
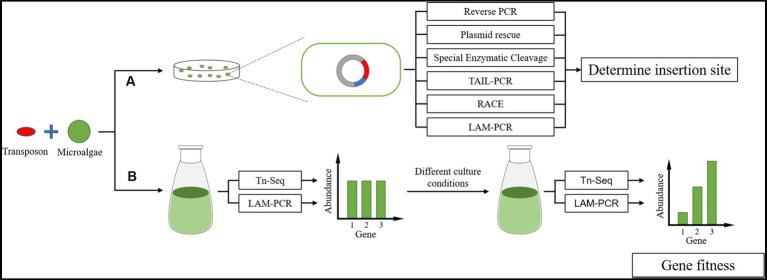
Transposon insertion site sequencing in microalgae. **(A)** Individual mutants with obvious phenotypes and research values are often sequenced using simple methods for equipment and operation. **(B)** High-throughput microalgae sequencing to establish a transposon insertion mutant library.

High-throughput sequencing will be the primary method for studying gene function in the future, based on the current research trend of microalgae. A large amount of transposon insertion site data will be obtained by high-throughput sequencing concerning gene function annotation or gene fitness to obtain gene expression in different growth environments. This becomes more of a need for methods that allow high-throughput determination of transposon insertion sites. However, using high-throughput sequencing methods becomes less necessary to determine transposon insertion sites for individual mutants with obvious phenotypes, especially from the point of view of costs. It is simple and easy to control costs using enzyme digestion and multiple primer amplification methods (Figure 9).

Fauser et al. (2022) determined the insertion site of each transposon by high-throughput sequencing using random transposon insertion into the genome of the model organism *C. reinhardtii*. These authors determined the phenotype of over 58,000 mutants by screening them under more than 121 different environmental growth conditions and chemical treatments. Fifty-nine percent of the genes in *C. reinhardtii* were represented by transposon insertion mutants that exhibited at least one phenotype. This is the most complete and comprehensive library of eukaryotic microalgal mutants known, providing a basis for the function of thousands of genes in *C. reinhardtii*. Previously, functionally unknown genes could be identified based on their functions, including DNA repair, photosynthesis, CO_2_ concentration mechanisms, and ciliogenesis.

Broddrick et al. (2016) used random transposon insertion to create a mutant library of prokaryotic microalgae in PCC 7942. These researchers identified genes essential for PCC 7942 under specific growth conditions through changes in the fitness of individual genes under different growth conditions. The genome-scale metabolic model of PCC 7942 was revised to produce a highly accurate metabolic model. Some previously unknown metabolic features of PCC 7942 were identified, including the nonessential nature of the TCA cycle.

## 6. Conclusion and future perspective

Transposon insertion marker DNA has become an essential tool for studying the functional genomics of organisms. A large number of DNA insertion lines and important mutations have been created in microalgae using this approach, which is necessary to determine the genomic sequence on either side of the insertion marker to identify genes tagged by transposon insertion. However, the sequences of the tagged genes cannot be obtained simply by conventional PCR reactions, which require a specific experimental design and technical methodological modifications. Current sequencing methods have distinctive characteristics and different problems. The reverse PCR and plasmid rescue methods are simple and operationally uncomplicated, with easily controllable costs but lower success rates. The special enzyme digestion method is more specific but due to the restrictive enzyme digestion sites, resulting in insufficient applicability. TAIL PCR and RACE technological steps have higher success rates than previous methods (Chen N. et al., 2016; Tan et al., 2019). Still, pre-processing LAM-PCR and Tn-Seq have high success rates and are especially suitable for high-throughput sequencing and establishing mutant libraries. However, the cost of sequencing a single mutant is high, and the amount of invalid data during sequencing is vast, which may be due to the lack of sufficient specificity of the sequencing primer used or the insufficient screening capacity of the available equipment for large amounts of data. These invalid data can be filtered in subsequent data processing, and generally will not cause errors in subsequent analysis and target selection. Therefore, we need to consider several factors when arranging sequencing experiments (e.g., experimental conditions, experimental schedule, cost, and the combination of multiple methods for sequencing and validation) to make reasonable and flexible experimental arrangements (Table 2).

**Table 2:** Comparison of sequencing methods.

Method		Advantages	Disadvantages
Enzymatic digestion	Reverse PCR	Simple operation, low cost.	The sequencing results of mutation sites are unstable and not suitable for high-throughput sequencing.
	Plasmid rescue	The results are not prone to false positives.	Not suitable for high-throughput sequencing.
	Specific enzymatic cleavage	The method is simple and supports high-throughput sequencing.	The short sequence used for sequencing leads to unstable results.
Multiple primer amplification method	TAIL PCR	Strong specificity, high efficiency.	Not suitable for high-throughput sequencing.
	RACE	Strong specificity, high efficiency.	Not suitable for high-throughput sequencing.
	LAM-PCR	Strong specificity, high success rate, suitable for high-throughput sequencing.	High cost.
Tn-Seq		Strong specificity, high success rate, suitable for high-throughput sequencing.	Long test cycle, heavy data processing workload.

The main future development direction will be improving the success rate and cost control to solve the problem of flanking sequencing after transposon insertion. It is currently difficult for all sequencing methods to reach a 90% success rate. A large amount of invalid data needs to be processed, even for high-throughput sequencing, which invariably raises the technical threshold and labor costs of the equipment. It is necessary to improve the transposon and sequencing methods to solve these problems. First, the transposon can be modified while retaining random insertion ability. The transposon itself should be able to carry a more easily identifiable tag, reducing the misoperation of devices in the amplifying process of target sequences and sequencing reads. Second, with technological and equipment updates, the accurate sequencing method is constantly updated, and sequencing costs decrease.

In conclusion, determining bipartite sequences after transposon insertion will be increasingly accessible, fast, and inexpensive with the development of various sequencing methods and transposon technologies. Applying the latest gene function research methods to microalgae can facilitate effective transformation and make them more excellent engineering microalgal chassis cells. Consequently, they can contribute better to human food, energy, and environmental protection.

## Author contributions

XH and QW conceptualized the idea for manuscript. XH, YF, CM, and HC drafted the manuscript. QW evaluated the manuscript and improved the content. All authors contributed to the article and approved the submitted version.

## Funding

This work was supported jointly by the National Key R&D Program of China (2021YFA0909600), the National Natural Science Foundation of China (32170138 and 31870041), the Natural Science Foundation of Henan Province (212300410024), the Program for Innovative Research Team (in Science and Technology) in University of Henan Province (22IRTSTHN024), and the 111 Project (#D16014).

## Conflict of interest

The authors declare that the research was conducted in the absence of any commercial or financial relationships that could be construed as a potential conflict of interest.

## Publisher’s note

All claims expressed in this article are solely those of the authors and do not necessarily represent those of their affiliated organizations, or those of the publisher, the editors and the reviewers. Any product that may be evaluated in this article, or claim that may be made by its manufacturer, is not guaranteed or endorsed by the publisher.
